# Catastrophizing and Risk-Taking

**DOI:** 10.5334/cpsy.91

**Published:** 2023-01-17

**Authors:** Alexandra C. Pike, Ágatha Alves Anet, Nina Peleg, Oliver J. Robinson

**Affiliations:** 1 Department of Psychology, University of York, York, YO10 5DD, GB; 2 York Biomedical Research Institute, University of York, YO10 5DD, GB; 3 Anxiety Lab, Neuroscience and Mental Health Group, Institute of Cognitive Neuroscience, University College London, London, GB

**Keywords:** Anxiety/anxiety disorders, CBT/Cognitive Behavioural Therapy, Cognition, Web-based

## Abstract

**Background:**

Catastrophizing, when an individual overestimates the probability of a severe negative outcome, is related to various aspects of mental ill-health. Here, we further characterize catastrophizing by investigating the extent to which self-reported catastrophizing is associated with risk-taking, using an online behavioural task and computational modelling.

**Methods:**

We performed two online studies: a pilot study (n = 69) and a main study (n = 263). In the pilot study, participants performed the Balloon Analogue Risk Task (BART), alongside two other tasks (reported in the Supplement), and completed mental health questionnaires. Based on the findings from the pilot, we explored risk-taking in more detail in the main study using two versions of the Balloon Analogue Risk task (BART), with either a high or low cost for bursting the balloon.

**Results:**

In the main study, there was a significant negative relationship between self-report catastrophizing scores and risk-taking in the low (but not high) cost version of the BART. Computational modelling of the BART task revealed no relationship between any parameter and Catastrophizing scores in either version of the task.

**Conclusions:**

We show that increased self-reported catastrophizing may be associated with reduced behavioural measures of risk-taking, but were unable to identify a computational correlate of this effect.

## Introduction

Catastrophizing is when an individual overestimates the probability that a catastrophe will occur: either an ‘objective’ catastrophe, which would be recognised by others as catastrophic (such as the death of a loved one), or a ‘subjective’ catastrophe, which is a negative event given undue importance by the individual (such as forgetting a meeting at work) (Pike, Serfaty, et al., 2021). Catastrophizing was first defined in relation to depression (Beck, 1963; Ellis, 1962), but is now recognised to be related to various aspects of mental ill-health (Austin & Richards, 2001; Bryant & Guthrie, 2005; Clark, 1986; Gellatly & Beck, 2016; Hazlett-Stevens & Craske, 2003; Jenness et al., 2016; Rachman, 1998a, 1998b) and is targeted by a treatment: decatastrophizing, which is a part of cognitive-behavioural therapy (Ingram & Kendall, 1987).

However, the cognitive underpinnings of catastrophizing have been little-studied, which precludes us from understanding how catastrophizing develops, how it is maintained, and which aspects of cognition should be targeted to reduce it. Understanding these factors may enable us to refine and target interventions, such as decatastrophizing, and potentially also develop preventative strategies.

In this study, we set out to investigate the specific cognitive processes associated with catastrophizing using a battery of cognitive tasks, including one measuring risk-taking. We used our previously developed self-report Catastrophizing Questionnaire (Pike, Serfaty, et al., 2021) to measure catastrophizing. This questionnaire has been shown to have good psychometric properties, and is specific to catastrophizing, unlike other self-report measures.

We anticipated that increased catastrophizing would be associated with reduced risk-taking. Specifically, we hypothesized that those who catastrophize will believe that the worst outcome is most likely in any situation where the outcome is uncertain: especially those that involve taking risks. This may lead to significant risk-aversion, above that seen in the general population. Notably, catastrophizing is thought to be strongly related to *minimization* (Beck, 1963), which is when individuals underestimate their ‘performance, achievement or ability’. This lack of self-belief might result in even greater reluctance to engage in risks: either because the individual believes they may make the wrong choice if the outcome of the risk is within their control, or because they do not believe they will be able to cope with the potential negative outcome. Overall, we propose that catastrophizing results in increased avoidance and heightened risk aversion.

Research has previously shown that risk aversion is elevated in anxiety (Charpentier et al., 2017; Giorgetta et al., 2012; Hartley & Phelps, 2012; Maner et al., 2007). We hypothesized that catastrophizing may drive the relationship between anxiety and risk aversion: catastrophizing is a construct which is elevated in anxiety, not normally measured or controlled-for in studies of anxiety and cognition, and is theoretically linked to risk aversion.

We used a modified balloon analogue risk task to measure risk-taking, and manipulated the cost of taking risks (i.e. level of punishment) by block to examine whether this would alter behaviour. Following an initial pilot study, we performed a larger scale investigation using this task. We also used computational modelling to allow us to better capture separate cognitive processes: such as the rate of updating a prior belief, risk-taking and decision noise. Fractionating these components of broader cognitive processes may allow further progress in understanding the ‘active ingredients’ of decatastrophizing therapy, and also allow us to design more specific interventions that target the precise aspects of cognitions that lead to the experience of catastrophizing.

In sum, to better understand the cognitive processes which relate to catastrophizing, we assessed the relationship between self-reported catastrophizing symptoms and risk-taking, in a pilot study and a larger main study. We also fit a series of computational models to task data and explored the relationship between these model parameters and catastrophizing, to understand how risk-taking computations may vary as catastrophizing levels change.

## Materials and Methods

Data, code and preregistered protocols are available at https://doi.org/10.17605/OSF.IO/Z2RGK (Pike, Alves Anet, et al., 2021). Ethical approval was given by the University College London Research Ethics Committee (reference: 15253/001). All participants gave informed consent.

### Procedure

We performed a pilot study (n = 69) to explore three candidate cognitive processes (note that we report the results for two of these tasks in the supplement, and focus on the results from the Balloon Analogue Risk Task in this paper). Subsequently, and based on an updated power analysis, we collected a larger sample of participants (n = 263) to examine the relationship between catastrophizing and risk-taking using the BART task. In both studies, participants completed six questionnaires measuring catastrophizing, anxiety, worry, depression, and the impact of the COVID pandemic (see supplementary material). After completing the questionnaires, participants were asked to perform either three cognitive tasks (pilot study), or just the BART task (main study). Completion of all the procedures took on average 30 minutes (pilot study) or 20 minutes (main study).

### Participants

We recruited 70 participants (pilot study) and 266 participants (main study), aged between 18 and 40 years, fluent in English, with no history of cognitive impairment, dementia, or impaired vision or hearing. We used the Prolific platform to recruit these participants (Palan & Schitter, 2018; https://app.prolific.co/) and tested them using Gorilla, an online experiment building platform (Anwyl-Irvine et al., 2020; https://gorilla.sc). The sample size for each study was determined using G*Power (version 3.1, Faul et al., 2009), see supplement. Participants were compensated for their time at a rate of £7.50/hour. Demographic information on participants was acquired through Prolific, and additional information on mental health diagnosis was acquired using a short series of questions presented in Gorilla. See supplement for exclusion criteria.

### Questionnaires

Participants completed five brief questionnaires to assess catastrophizing, anxiety, trait anxiety, worry and depression. These were, respectively: the Catastrophizing Questionnaire (CQ; Pike et al., 2021), the Generalised Anxiety Disorder Assessment (GAD-7; Spitzer et al., 2006), the State-Trait Anxiety Inventory (STAI-T; Spielberger et al., 1983), the Penn State Worry Questionnaire (PSWQ; Meyer et al., 1990) and the Patient Health Questionnaire (PHQ-8; Kroenke et al., 2009; Shin et al., 2019). We also collected data on the effects of the COVID-19 pandemic (see supplement) on our participants due to the timing of our data collection (April–July 2020), and because this pandemic is thought to have systematically impacted mental health (Dubey et al., 2020; Lee, 2020; Vindegaard & Benros, 2020).

### Balloon Analogue Risk Task

**Figure 1:**
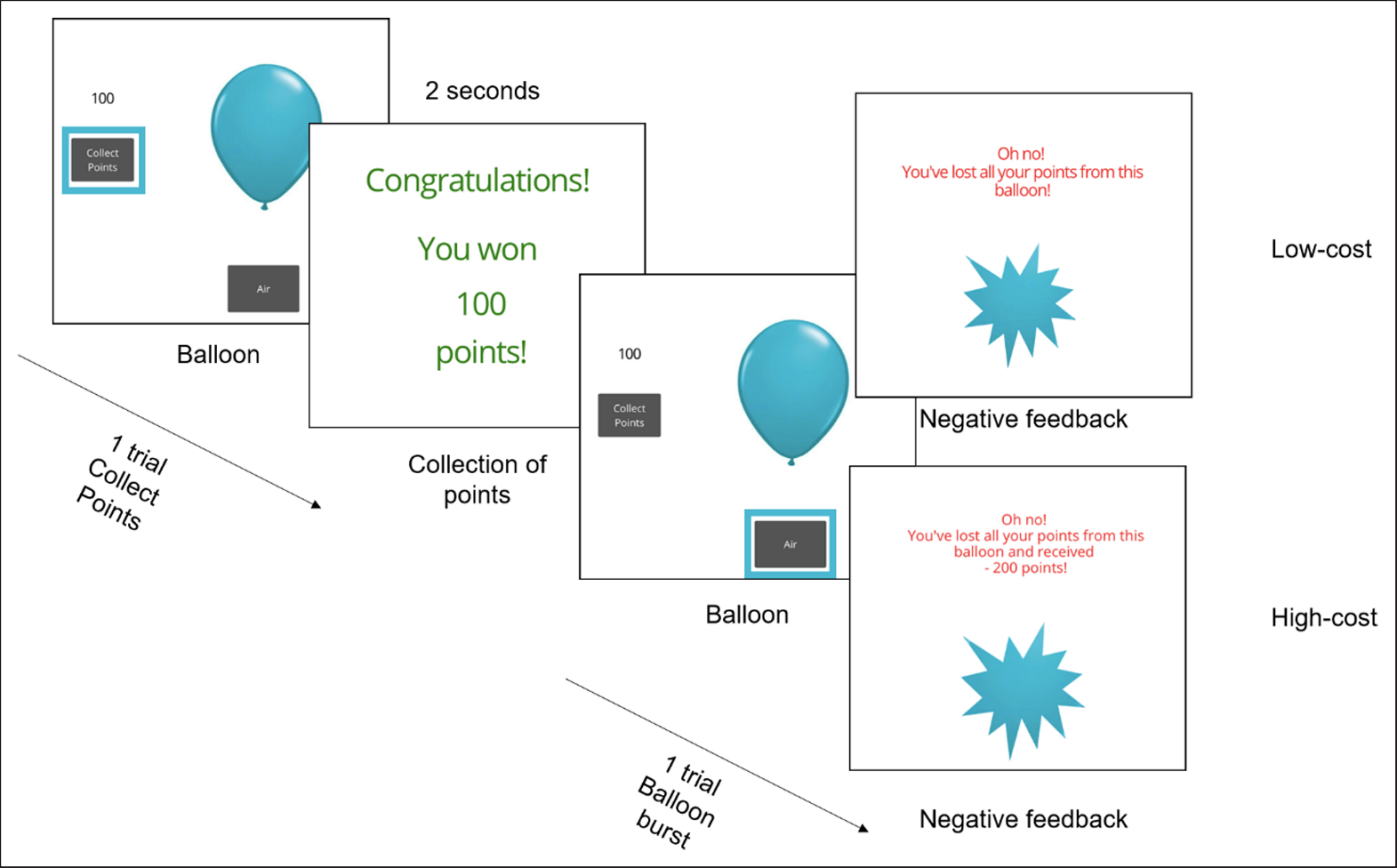
Our modified BART task required participants to press a button labelled “Air” (or, in the main study, use a keyboard press) to pump up a balloon and earn points. The balloon grew in response to every pump. The task was divided into two blocks, each with 30 trials (30 balloons). Participants were told how far through each block they were (e.g. Balloon 1 of 30). Participants were instructed to pump the balloon as many times as they wished and to collect their points at any time, but were warned that the more they pumped the balloon up, the more likely it was to burst. This burst was associated with a penalty – either the loss of all the points for that balloon (low-cost block), or all the points for that balloon and an additional 200 points (high-cost block, pilot study) or 1000 points (high-cost block, main study). Once they had chosen to collect the points, they were presented with a screen containing the number of points they had earned from that balloon (see ‘Point Collection’ screen), or if the balloon burst, they received the ‘Negative Feedback’ screen that corresponded to the block they were in.

To measure risk-taking, participants performed a modified version of the Balloon Analogue Risk Task (BART; Figure 1; Lejuez et al., 2002). In this task, participants were told to try to win points by pumping the balloon up, and that every pump increased their points but also increased the risk of the balloon bursting. If the balloon burst, they would be faced with a penalty. In both blocks (high-cost and low-cost) this penalty consisted of losing all points accumulated by pumping that specific balloon, however in the high-cost block, a set number of additional points were deducted. Participants started initially with 0 points, and with each pump they gained 10. Trials ended either when the participant pressed the ‘collect points’ button, or the balloon burst. If they collected their points, they won however many points they had gained in that trial; if they pressed the ‘air’ button so many times that the balloon burst, they won no points. They were shown their total points at the end of each trial. Each balloon would burst after a certain number of pumps – this number was picked from an array which was randomly shuffled for each participant. The mean numbers of pumps needed to burst the balloon were 12.37 ± 3.49, with a range of 6–18 (LC block, pilot study only) and 12.23 ± 4.72, range 1–19 (all other blocks: HC in both studies, and LC block, main study- see Supplementary Figure 9).

In the pilot study, a ‘burst’ in the HC block resulted in an additional loss of 200 points. In the main study, we increased the penalty for bursting the balloon in the HC block (from 200 to 1000 points), as in our pilot study the block manipulation largely did not impact participant responses. Additionally, responses were made by keypress rather than on-screen buttons, to allow more rapid and accurate responding. This task has been made openly available at https://gorilla.sc/openmaterials/118145.

### Statistical Analyses

Data were analysed using R software (R version 3.6.1), and analysis largely took the form of correlation analysis. Diagnostic plots of correlation or regression residuals were inspected to ensure that the residuals were normally distributed. When data were negatively skewed, they were transformed using a square root transformation (sqrt(max(x)–x)), and when positively skewed, they were transformed using an equivalent square root transformation (sqrt(x)). Where data that required transformation included negative numbers, a scalar was added so that all numbers were greater than 0 before square root transformation. In cases where no appropriate transformation could be found, non-parametric tests were performed instead.

#### Model-free analysis

We used a correlation test to understand the relationship between the number of pumps in the BART task and the Catastrophizing scores in each block. We also assessed the correlation between Catastrophizing scores and the per-participant difference in the number of pumps for each block and performed a mixed model with cost as a within-subjects factor to determine if the penalty was a moderator of the relationship.

In the supplement, we present results examining whether catastrophizing mediated the relationship between anxiety (a latent variable comprised of all three anxiety questionnaires) and the transformed number of pumps in the main study. We ran this mediation analysis using the ‘lavaan’ package in R, version 0.6–11. We also ran a more detailed structural equation model including latent variables for the other mental health constructs we measured, to examine specificity. Finally, in the supplement we also present the results of a correlation analysis and a longitudinal paired-samples t-test on the change in Catastrophizing Questionnaire scores pre-pandemic and mid-pandemic on our pilot participants, to explore the impact of COVID-19 on catastrophizing in this dataset.

#### Computational models

We also designed and fit computational models to participants’ performance on the BART task, in order to take advantage of trial-by-trial information and attempt to parameterise risk-taking more accurately than would be possible using a summary statistics approach.

We fit all models using the hBayesDM package (v1.0.2, https://rdrr.io/cran/hBayesDM/man; Ahn et al., 2017) and RStan (2.21.2, http://mc-stan.org/). All models used a non-centered parameterization, in which the parameters are drawn from a normal distribution which is written with a group mean for that parameter, a group standard deviation, and then a participant-specific error term. The priors for the group means and the error terms were drawn from a standard Normal distribution. The group standard deviation was drawn from a Normal (0,0.2) distribution. Parameters which were bounded were then subsequently transformed: learning rates using an approximate Phi transformation to bound them in the interval [0,1], and other parameters using an exponential transform to bound them above 0. We performed sampling with four chains and 3000 iterations (of which 2000 were warm-up) using Markov-Chain Monte-Carlo. We compared the fit of these models using the integrated BIC (Huys et al., 2011, for justification see supplement), and also required that the best-fitting model had good recovery (correlations between synthetic and recovered parameters of *r* > 0.6, no significant trade-off between parameters of *r* > 0.4) and then performed Pearson’s correlations between the resulting risk-taking parameter from each block and participants’ Catastrophizing scores.

#### Model specification

We fit the two models included within the hBayesDM package – the classic four-parameter model (van Ravenzwaaij et al., 2011), and an exponential-weight mean-variance model (Park et al., 2019). We also fit a set of models that we designed based on prospect theory (Kahneman & Tversky, 1979), that allowed participants to update the number of pumps they planned to make within each trial, rather than at the end. The corresponding model equations can be seen in the supplementary material. Subsequently, we tested models that were modified from the best-fitting one of these models (which was the classic four-parameter model) – with all possible combinations of the included parameters (15 models in total).

The full four-parameter model included the following parameters: ‘risk taking’, which represents participant’s tendency to prefer a risky option when values are equivalent; ‘learning rate’, or the extent to which participants updated their prior estimate of the probability of the balloon bursting by the outcome of each trial; ‘prior belief’, which is participants’ initial expectation of the balloon bursting; and ‘inverse temperature’, which governs choices that don’t align with the values estimated by participants. The participant, on each trial (or balloon, denoted *t*), begins with a belief that the balloon will burst ( \[p_t^{belief}\]), which is constant throughout that trial. After each trial, the belief is updated:

Eq. 1 \[p_t^{belief} = 1 - \frac{{priorBelief + learningRate*n\_successes}}{{1 + learningRate*n\_pumps}}\]

Where *n_pumps* represents the number of pumps the participant made on that previous trial, and *n_successes* is the number of pumps on the previous trial that didn’t lead to an explosion (i.e. *n_pumps* –1 if there was an explosion, *n_pumps* otherwise), and *learningRate* is a parameter in the interval [0,1] that is estimated separately for each participant. The participant determines the optimal number of pumps on each trial (*number_t_*, which is also constant throughout the trial) based on their tendency to take risks (*riskTaking*), and the probability that pumping the balloon up will make it burst:

Eq. 2 \[numbe{r_t} = \frac{{ - riskTaking}}{{{\mathrm{ln}}(1 - p_t^{belief})}}\]

The actual probability that they will pump up the balloon at each pump opportunity (*opportunity*) within each trial (*t*) depends on the value of *number_t_*, which can be considered to be the number of pumps that they think is appropriate on that trial, along with their behavioural consistency or inverse temperature, entered into a sigmoid equation:

Eq. 3 \[p_{t,opportunity}^{pump} = \frac{1}{{1 + {e^{inverseTemperature(opportunity - numbe{r_t})}}}}\]

## Results

Participant demographic variables for both studies are reported in the Supplementary Material. In brief, the age range was 18–41, and approximately 40% of the sample were female. Slightly over 50% of the sample were students, and their mean prolific score was over 99/100 (an indication that they generally produce good quality data that researchers do not have cause to reject). 21% of participants reported having been diagnosed with a mental health condition.

### Pilot Study

#### Model-free analysis

**Figure 2:**
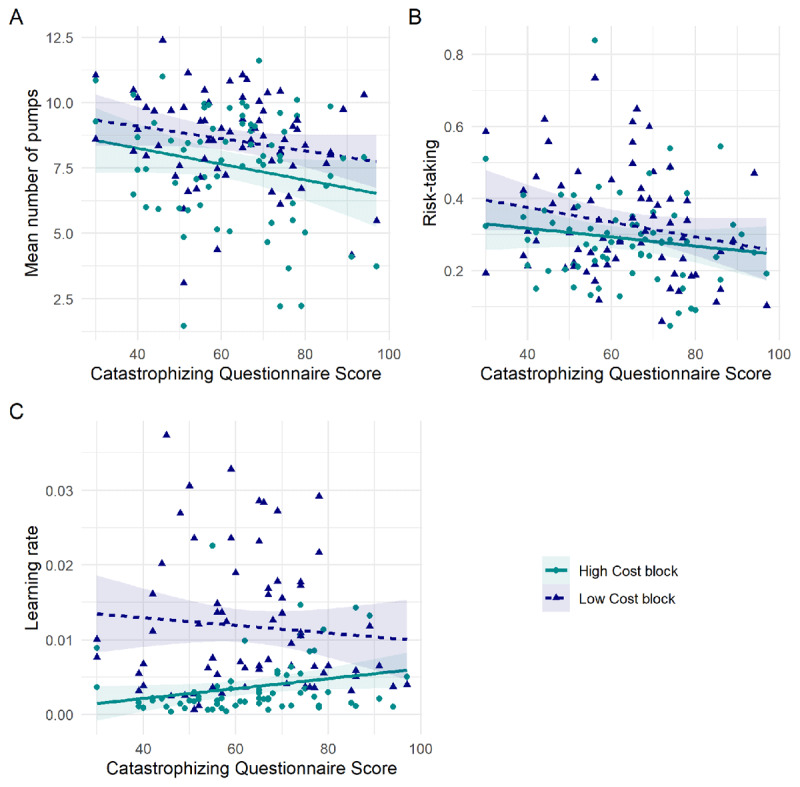
Relationships between measures derived from the BART task and Catastrophizing scores in the pilot study (x axis), displaying the per-participant mean on each variable, and with a regression slope fitted using the ‘lm’ method from ggplot2. **A)** There was no significant relationship between the transformed number of times each participant pumped the balloon up and their Catastrophizing scores, in either block (LC or HC) of the BART task. **B)** There was no significant relationship between Catastrophizing scores and risk-taking in a computational model of the BART task. **C)** There was a significant relationship between Catastrophizing scores and learning rate in a computational model of the BART task, but only in the ‘high-cost’ block.

We calculated the mean number of times each participant pressed the ‘Air’ button per balloon, on trials in which the balloon did not burst. There was no correlation between the Catastrophizing scores and the transformed mean number of pumps in either block (LC block: *r*_67_ = –0.222, *p* = .067; HC block: *r*_67_ = –0.208, *p* = .086 Figure 2A). However, there was a relationship between Catastrophizing Scores and the transformed mean number of pumps across both blocks (*r*_67_ = –0.242, *p* = .045).

#### Computational analysis

The best-fitting model was the full four-parameter model, with the following parameters: prior belief about the number of pumps before a balloon burst, learning rate, risk-taking, and an inverse temperature parameter. This model was better than the exponential-weight mean-variance model, with a Bayes Factor of 2.05 (see Supplement), and substantially better than the next best-fitting model within the four parameter family, which was identical except with a fixed effect of belief rather than a free parameter (Bayes Factor 5.74). Notably, there was no relationship between risk-taking and Catastrophizing Questionnaire scores (low cost block: *r*_67_ = –0.211, *p* = 0.082; high cost block: *r*_67_ = –0.147, *p* = 0.227; Figure 2B). However, there was a significant correlation between Catastrophizing Questionnaire scores and learning rate in the ‘high-cost’ block (*r*_67_ = 0.246, *p* = 0.042; Figure 2C). No other correlations reached statistical significance (see Supplement). To further examine this relationship, we replicated this study in a larger sample.

### Main Study

#### Model-free analysis

**Figure 3:**
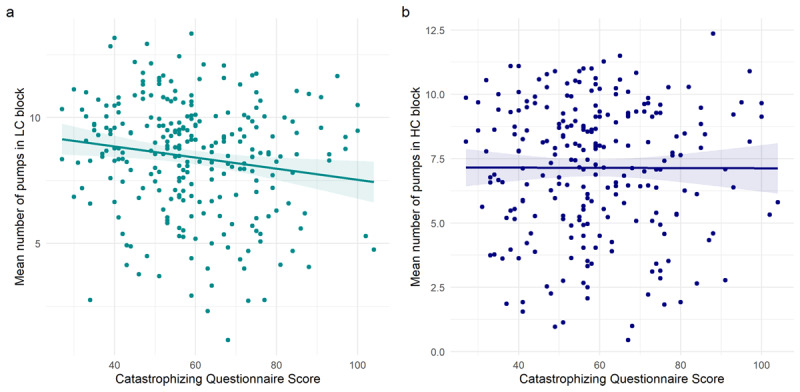
Relationships between measures derived from the BART and Catastrophizing scores in the main study (x axis), displaying the per-participant mean on each variable, and with a regression slope fitted using the ‘lm’ method from ggplot2. **A)** A significant negative correlation between the transformed mean number of pumps in the LC block of the BART task and Catastrophizing scores. **B)** No correlation between the transformed mean number of pumps in the HC block and Catastrophizing scores.

In the main study, we further examined the relationship between catastrophizing and risk-taking using the BART task. 263 participants performed the low-cost block, of whom 242 also performed the high-cost block. The different blocks elicited different behaviour: there was a significant difference in the mean number of pumps in each block (low cost mean = 8.44 (sd = 2.25), high cost mean = 7.14(2.56), *t*_474.34_ = 5.97, *p* < 0.001). We found a significant correlation between Catastrophizing scores and the transformed mean number of pumps in the LC block (*r*_260_= -0.156, *p* = .012, Figure 3A), but not the HC block (*r*_237_ = 0.001, *p* = .979, Figure 3B). To assess whether this significant difference was itself significant, we evaluated whether there was a correlation between the transformed difference in the number of pumps between the LC block and the HC block and the Catastrophizing scores. This was not significant (*r*_237_ = –0.124, *p* = .057). Notably, cost level was a moderator of the effect of number of pumps on catastrophizing in a mixed model including cost, Catastrophizing score, and a random intercept of participant (F_1,243.73_ = 4.45; *p* = 0.036).

We also performed a post-hoc analysis to assess whether catastrophizing had an effect by inhibiting participants’ tendency to pump the balloon up on trials after a ‘burst’ outcome. We ran two mixed models, one for each block, with two factors: previous burst (whether the outcome on the previous trial was an explosion) and Catastrophizing score, with a random effect of participant. There were no main effects of previous explosion or of Catastrophizing score, and no significant interaction effects (all *p* > 0.3).

#### Computational analysis

**Figure 4:**
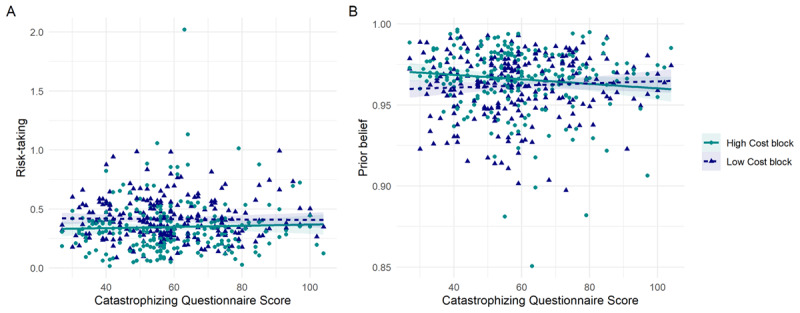
Relationships between computational parameters derived from the BART and Catastrophizing scores in the main study (x axis), displaying the per-participant mean on each variable, and with a regression slope fitted using the ‘lm’ method from ggplot2. **A)** No significant relationship between the risk-taking parameter and Catastrophizing scores. **B)** No significant relationship between the prior belief parameter and Catastrophizing scores.

The best-fitting model for the main task data was the same as in the pilot study: the classic four-parameter model, with a prior belief term, learning rate, risk-taking term and inverse temperature term. This was better than the next best model, with a Bayes Factor of 6.8. There was no correlation between risk-taking and Catastrophizing Scores (low cost block: *r*_236_ = 0.016, *p* = 0.809; high cost block: *r*_236_ = –0.037, *p* = 0.575; Figure 4A). There was also no significant relationship between catastrophizing and any parameter, with the lowest *p*-value 0.088 (for the relationship between prior belief and catastrophizing in the high-cost block: *r*_235_ = –0.111, *p* = 0.088; Figure 4B). The correlations between all parameters and Catastrophizing scores can be seen in the Supplement. Notably, cost level was not a moderator of the effect of modelling parameter risk-taking on catastrophizing in a mixed model including cost, Catastrophizing score, and a random intercept of participant (F_1,236_ = 0.493; *p* = 0.483).

When we added in the computational model parameters to a structural equation model, none of these were significantly related to Catastrophizing scores.

## Discussion

In this paper, we demonstrate for the first time a correlation between self-reported catastrophizing and a behavioural measure of risk-taking. In the main study we found a significant negative correlation between the number of pumps of the balloon participants made in the ‘low-cost’ block of our modified BART task, and Catastrophizing scores. This finding suggests that individuals who catastrophize may find the risk of the balloon bursting more intolerable, and are driven to avoid this outcome. Simply put, increased catastrophizing is associated with reduced risk-taking behaviour. When we analysed this finding further using computational models of the decision-making process, we were unable to identify a relationship between any model parameter and Catastrophizing scores in either block.

Reduced risk-taking has been seen previously in anxiety (Charpentier et al., 2017; Giorgetta et al., 2012; Hartley & Phelps, 2012; Maner et al., 2007). Risk-taking can be adaptive, and potentially necessary to achieve a desired result (e.g. taking a flight to your holiday abroad). Reduced risk-taking, however, may result in individuals with anxiety learning less about risks, perhaps increasing avoidance in general, which is a common feature in anxiety (Barlow, 2004). We hypothesized here that reduced risk-taking in anxiety may be driven (at least in part) by increased catastrophizing in anxiety disorders. Specifically, if one assumes that the outcome of any decision made tends to be catastrophic, this discourages risk-taking. Our mediation analysis (Supplement) did not show this: we did not observe a relationship between anxiety and risk-taking. This suggests that either our task was insufficiently sensitive to risk-taking, or perhaps that our general population sample did not have sufficient levels of anxiety for a relationship to be detected. Alternatively, it could be that initial findings of altered risk-taking in anxiety are not robust, or perhaps depend on moderators that we did not measure here.

It is surprising that we only observed a relationship between catastrophizing and risk-taking in one block of our task in the main study, especially considering that in the pilot study the relationship was of similar magnitude in each block. This may be a consequence of the modifications we made to the ‘high-cost’ block between the studies, particularly the increase of the penalty for balloon-bursting. This could have resulted in a ‘floor effect’, where all participants found the costs of the balloon bursting sufficiently high that they preferred not to pump the balloon up at all. However, after further inspection of the data we found no floor effect in either block (Supplementary Figure 9).

Interestingly, the relationship between risk-taking and catastrophizing was not reflected in our computational model of the task. There was no relationship between any parameter in the model and Catastrophizing scores in the ‘low-cost’ block – perhaps indicating that the number of times participants choose to pump the balloon is not well explained by this model, or that several processes coincide to produce this result, rather than it arising simply out of an alteration in one aspect of cognition. In particular, our *model* parameterization of ‘risk-taking’ seems not to have captured or fully explained the change in risk-taking *as operationalized by the task* in this block. Alternative models (such as the exponential-weight mean-variance model or models based on prospect theory) fit the data less well, so could not be used.

Our findings suggest that basic performance on risk-taking tasks (i.e. number of pumps in the BART task) could provide a translational endpoint for potential interventions to reduce catastrophizing. In other words, efficacy of treatments for catastrophizing could be assessed by demonstrating their ability to increase risk-taking. Behavioural measures of such constructs have a core advantage over assessment based on self-report measure, as they can also be assessed in translational non-human models and as a result can be used to screen pharmaceutical interventions and probe underlying neurobiology (Pike, Lowther, et al., 2021).

Risk-taking inherently involves an understanding of the probabilities of both the risk and the reward, and the ability to weigh up the costs and benefits of each of these. Individuals who catastrophize may perceive the probability of a negative outcome as higher, whether this is in a risky situation, or in one that is simply uncertain. Therefore, the expected value of a risky option may be reduced, which may result in apparent risk-avoidance. Future work could use prospect theory models in specifically designed prospect-theory tasks to understand the extent to which the perception of probabilities, particularly of low-probability but high-cost outcomes, might influence individuals’ tendency to catastrophize. Prospect theory paradigms may also allow us to disentangle whether risk- or loss-aversion is related to catastrophizing: the data from the BART task could be explained by either (though note other research indicates anxiety is associated with risk aversion rather than loss aversion, Charpentier et al., 2017).

## Limitations

Our modified BART task included two different levels of penalty for bursting the balloon, which we anticipated might alter the relationship between catastrophizing and risk-taking. Including a range of intermediate costs in future studies might allow better and more nuanced characterisation of this potential moderating effect. Furthermore, greater characterisation of this relationship would also be aided by research investigating whether the relationship between catastrophizing and risk-taking is context-general or context-specific. Studying catastrophizing in relation to specific domains of risk-taking, e.g. social risk-taking, or health risk-taking, might further clarify the bounds of this relationship.

All data presented in this paper was collected using online testing, which may introduce noise. However, the recruitment platform we chose to use, Prolific, has been shown to produce better quality data than other platforms (Peer et al., 2017), and may also result in more diverse participants than can be recruited in-person (Peer et al., 2017). Of note, our sample of participants had high ‘prolific scores’ (Supplementary Table 2) – a measure of their performance on the platform (Palan & Schitter, 2018).

Finally, our results may have been confounded by the global COVID-19 pandemic, as health-related anxiety is an important feature of catastrophizing (Salkovskis & Warwick, 1986). We present results in the supplement suggesting that, although there was a relationship between worry about COVID-19 and catastrophizing, participants’ Catastrophizing scores did not change when comparing pre- and during- the pandemic. We, therefore, hypothesise that whilst those who catastrophize may also have been more concerned about the pandemic, participants’ level of catastrophizing remained stable: suggesting that our results are not driven by the effects of the pandemic.

## Conclusions

In conclusion, we demonstrate a relationship between catastrophizing as assessed by a self-report questionnaire and risk-taking as assessed by a non-subjective cognitive task. Specifically, greater catastrophizing is associated with reduced risk-taking in a version of the BART task where the cost of taking a risk is relatively low. However, there were no relationships between catastrophizing and computational model parameters of this task. Importantly, risk-taking could therefore act as a translational endpoint for catastrophizing interventions.

## Data Accessibility Statement

The data that support the findings of this study are openly available on the Open Science Framework (OSF) at https://doi.org/10.17605/OSF.IO/Z2RGK.

## Additional File

The additional file for this article can be found as follows:

10.5334/cpsy.91.s1 Supplemental Material. Supplementary methods, results and references.
