# Supplementary material for: Catastrophizing and Risk-Taking
Source: Comput Psychiatr. 2023 Jan 17;7(1):1–13. doi: 10.5334/cpsy.91 (PMC11104403; doi:10.5334/cpsy.91)
Supplement: Supplemental Material. — Supplementary methods, results and references. [file cpsy-7-1-91-s1.pdf]

Supplement

1 Supplementary Methods

1.1 Power Calculation

For the pilot study a sample size of 63 participants was required to achieve 95% power (at alpha=.05) and to detect effect sizes of over *r*=.4 (a minimally interesting effect size in this exploratory work) in a Pearson's correlation test between Catastrophizing Questionnaire scores and any of the behavioural outcome measures. For the main study a sample size of 269 participants was required to achieve 80% power (at alpha = .05) to detect an effect size of *r*=-0.17 in a test for the correlation between mean pumps in the BART task and catastrophizing score.

1.2 Exclusion criteria

In the Pilot study, one participant was excluded as they responded by giving random numbers in the Mathematics task. In the Main study, due to a technical error, only 242 had data for both blocks. We excluded three participants who did not pump the balloon up at all in one or both blocks of the task, and therefore had n=263 for the low cost block, and n=239 for the high cost block.

1.3 Model Comparison and the use of Integrated BIC

The use of information criteria to adjudicate between different models is the subject of substantial debate within the field. All methods have their own limitations, and we use the integrated BIC as the one that has the least issues given our dataset and the models we wish to compare.

The developers of Stan recommend the use of WAIC and LOOIC (Vehtari, Gelman, & Gabry, 2017), as they are fully Bayesian rather than reliant on point estimations. They also argue against the use of DIC, which is common in other modelling tools, such as BUGS. One of their particular concerns is that DIC can produce negative estimates of the number of parameters in a model. However, when the authors trialled the use of WAIC and LOOIC in this dataset, a high proportion of the Pareto k diagnostic values were high, suggesting that the assumptions for the smoothed importance sampling used to calculate these criteria are not met. Relatedly, we tend to observe that the estimate of parameters in a model for models with high numbers of parameters (>1 per person, as is common in computational neuroscience models of learning or decision making) tend to be very inflated. For example, in the models used in this paper, the maximum number of effective parameters was 944131.3, far more than the number of parameters multiplied by the number of participants. We therefore use integrated BIC as the best option available to us, given the above considerations.

1.4 Catastrophizing Questionnaire

The design and psychometric properties of this questionnaire have been reported elsewhere (Pike, Serfaty, & Robinson, 2021). However, as this questionnaire is relatively recent, we present the items here for the readers' convenience.

Catastrophizing questionnaire

Please indicate how often over the last two weeks the following statements have applied to you. These items are rated on a Likert scale with the items never, rarely, sometimes, often and always.

If I have a problem, I wish somebody else would take the burden away from me.
I think about all the ways that things can go wrong.
I imagine that I might have a serious health issue.
I think about things that others would say are unlikely to happen.
If I have an exam, I think that if I fail it will affect my whole future.
I think that we are facing a major environmental disaster that humankind will not survive.
I think that a disaster is going to happen to me.
If I have a disagreement with a person I care about, I think that we will not make up.
I overthink and then become unable to decide what to do.
I think I am going to make a big mistake soon.
If I have a medical symptom (headache, heart palpitations, stomach ache), I think I must have a serious disease.
If I have an illness, I don't believe that treatment will work.
If I text a friend and they don't message me back, I immediately think that they're upset with me.
I think that any problem will only get worse as time passes.
If my partner is late home from work, I think that they have been in an accident.
I think that what I am going through is much worse than what others have experienced.
I think I am going to lose someone close to me forever.
I think that I will always have money problems.
I think that we will see another world war in the next few years.
If I have a bad month at work, I think that I will get fired.
I think that the worst case scenarios are very likely to happen.
I think that my house will be burgled.
I think about what will happen if I make a mistake.
I think that I am not very good at finding ways to solve my problems.

This questionnaire includes examples of catastrophizing that are relevant to different situations and contexts. However, the reader should note that during the development of this questionnaire catastrophizing did not appear to be highly context-dependent, as all of these items loaded onto a single factor when we performed factor analysis (Pike et al., 2021).

1.5 Prospect Theory Models

The prospect theory models that we designed and fit to our data had up to five parameters, with similar interpretations to the four parameter model: 'risk taking', which represents participant's tendency to prefer a risky option when values are equivalent; 'learning rate', or the extent to which participants updated their beliefs; 'prior belief', which is participants' initial expectation of how many pumps it will take before the balloon bursts; 'inverse temperature', which governs choices that don't align with the values estimated by participants. These models also included an additional parameter, 'loss aversion', which captures participants' sensitivity to losing the point they had accumulated. We fit models with all possible combinations of these five parameters to our data.

Participants' decision to pump the balloon up was estimated not only per trial *(t)*, but within each pump decision within that trial *(u)*.

Firstly, in models with the learning rate parameter, if the pump the participant was about to make would have been greater than their current belief of how many pumps it would take to burst the balloon, the 'pumpBelief' quantity was updated according to:

$$pumpBelief_{t,u} = pumpBelief_{t,u-1} + learningRate * (u - pumpBelief_{t,u-1})$$ *Supplementary Equation 1*

Subsequently, for every pump *u,* the prior belief of the number of pumps it would take for the balloon to burst was converted within the model into the probability the balloon would burst on the next pump*):*

$$probabilityBurst_{t,u} = \frac{1}{pumpBelief + 1 - u}$$ *Supplementary Equation 2*

Note that *probabilityBurst* was bounded between 0 and 1: when *pumpBelief* was greater than u+1 (i.e. the next pump would exceed the participants' current estimate of how many pumps it would take to burst the balloon, even after the learning step) *probabilityBurst* was set to 1. The pump-wise gain and loss values were then calculated (note that in models without loss aversion, valueLoss was just u-1). Note that these values reflect the actual scoring of the BART task: if you pump the balloon up and it doesn't explode you can then collect your points, which are proportional to the number of pumps you have completed on that trial, and if it does explode you cannot collect these points, equivalent to losing the number of points you had on the previous pump.

$$valueGain_{t,u} = u$$
$$valueLoss_{t,u} = (u-1) * lossAversion$$ *Supplementary Equation 3*

These quantities were then entered into an equation to calculate the expected value of pumping the balloon up. Note here that risk taking is a power function of the total uncertainty in the expected value: the multiple of the two possible outcome probabilities. This reflects the power curves used in the original prospect theory work.

$$\begin{aligned} expectedValue_{t,u} \\ &= \left(1 - probabilityBurst_{t,u}\right) * valueGain_{t,u} \\ &- probabilityBurst_{t,u} * valueLoss_{t,u} \\ &- (\left(1 - probabilityBurst_{t,u}\right) \\ &* \left(probabilityBurst_{t,u}\right))^{riskTaking} \end{aligned}$$ *Supplementary Equation 4*

Finally, the expected value was converted into the probability that the participant actually pumped the balloon up.

$$p_{t,u}^{pump} = \frac{1}{1 + e^{(inverseTemperature * expectedValue_{t,u})}}$$ *Supplementary Equation 5*

All parameters were constrained to be greater than 0; learning rate was additionally constrained to be in the interval [0,1].

1.6 COVID-19 Impact Questions

Due to the context in which we collected this data, we asked participants a series of questions about their experiences of the COVID-19 pandemic in both the pilot and main studies. These questions were as follows:

**Order: pilot**	**Order: main**	**Question**	**Response**	**Item included in COVID-worry measure?**

study	study			
**1**	6	How worried are you about the novel coronavirus (COVID-19) outbreak?	Slider – not worried at all to very worried	Yes
**2**	7	How likely do you think you are to catch the virus?	Slider – very unlikely to very likely	Yes
**3**	8	How do you think your health will be affected if you do catch the virus?	Slider – not at all to substantially	Yes
**4**	9	How likely do you think it is that a loved one will catch the virus?	Slider – very unlikely to very likely	Yes
**5**	10	If any of your loved ones does catch the virus, how do you think their health will be affected?	Slider – not at all to substantially	Yes
**6**	2	Are you in an at-risk group (according to your country)?	Yes/No	
**7**	3	Are any of your loved ones in an at-risk group (according to your country)?	Yes and I live with them; Yes but I don't live with them; No	
**8**	11	How do you think you will be affected by the global effects of the virus (for example economic recession, reduced health capacity)?	Slider – not at all to substantially	
9	**12**	**Instruction: Please answer the following questions according to your thoughts and behaviours in the last week.**		
**10**	13	How much is the following statement true: "I wash my hands more often and longer than necessary".	Slider – not true to very true	
**11**	14	How much have you been social distancing?	Slider – 'Not at all, I have been living my life as usual' to 'The only face-to-face contact I have is with people I live with (if you live with someone)'	
**12**	15	How would you rate your social-distancing experience?	Radio buttons – very good, moderately good, slightly good, slightly bad, moderately bad, very bad, not applicable	
**13**	16	How many times in a day do you think about the outcomes of the coronavirus outbreak?	Rating scale - None, 1 to 3, 4 to 6, 7 to 9, 10 to 12, 13 to 15, 16 or more	
**14**	17	How many months do you think the measures taken by your government (e.g.	Rating scale – 1 to 2, 3 to 6, 7 to 12, 12 or more	

		lockdown, social distancing) will last for?	
**15**	5	What is your current employment status?	Radio buttons – unemployed or job seeking, employed full-time not as a healthcare worker, employed full-time as a healthcare worker, employed part-time, self-employed, student, retired, home-maker full-time, other (please specify)
**16**	4	How many people live in your house at the present moment (including you)?	Dropdown – 1, 2, 3, 4, 5, 6, 7, other (please specify)
**17**	1	Do you have any flu-like symptoms?	Radio buttons – I don't have any symptoms, cough, fever, headache, tiredness, other (please specify)

Supplementary Table 1: Items and possible responses to the COVID-19 impact questionnaire. We also show the order these items were presented in the pilot and main study. We also show which items were summed to use as a measure of 'COVID worry'.

These questions were reordered for the main study, as is shown in the table above. The question about household size was changed to radio button responses, with options of 1, 2, 3, 4, 5, 6+, and employment. The item asking participants to rate their social-distancing experience was changed to a slider, from 'very bad' to 'very good'. The item asking how often participants think about the outcomes of the coronavirus outbreak, was also changed to a slider, with extremes of 'never' and 'over 15 times a day'. Item 14/17 was rephrased to 'How much longer do you think social distancing measures taken by your government will be in place for?', and participants responded on a slider from 'A few more weeks' to 'A year or more'.

We examined the relationship between the Catastrophizing scores and the scores in the COVID-19 impact questionnaire using a Pearson's correlation. We also analysed the difference between the Catastrophizing scores from the same participants at two time-points (July 2019 and April 2020) using paired t-tests to understand if participants' catastrophizing scores were affected by the COVID-19 pandemic, which could have confounded our results.

1.7 Other Tasks

In the pilot study, we included two other tasks alongside the BART task (reported in main paper). These tasks are publically available on Gorilla Open Materials (Cards: https://gorilla.sc/openmaterials/118148; Mathematics: https://gorilla.sc/openmaterials/117873;).

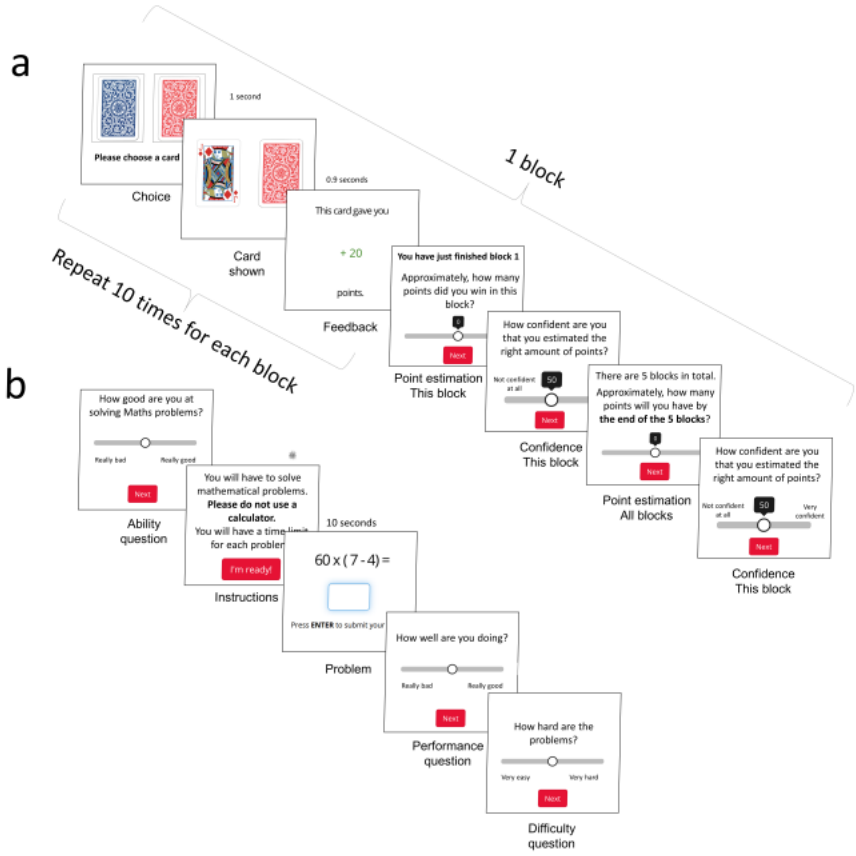

Supplementary Figure 1: Diagrams (not to scale) of tasks completed by participants in the pilot study. A) During the cards task, participants were instructed that they would be asked to choose between two cards presented to them, which could either give them points or take some away. They were informed that the aim of the task was to obtain as many points as possible, and that they should try to keep track of their points in their head. Subsequently, two cards (one red and one blue) were presented, and, after making a choice, participants were shown the number of positive or negative points that they had earned for that particular chosen card. Notably, in the second ten trials (trials 11-20) all the cards, regardless of the colour/side selected, resulted in negative points. Each card picked was classified as one trial and the total task consisted of 50 trials. Every ten trials participants were asked "How many points did you win in this block? You may estimate positive or negative points." and "How many points will you have by the end of this task? You may estimate positive or negative points", which they could answer by using a slider (ranging from -600 to +600). At this stage, participants were also told how far through the task they were. Participants were also asked how confident they were in their estimation of their current score and their total final score, to which they could answer on a slider ranging from 'very confident' to 'not confident at all'. B) Mathematics task. Participants responded using a slider to the question 'How good are you at solving Maths problems?', which ranged from 'really bad' to 'really good'. This was followed by an instruction screen, asking them to solve mathematical problems with a time limit without using a calculator. Problems were then presented for 10s, and every five trials, participants were asked 'how well are you doing?', which they responded to using a slider ranging from 'really bad' to 'really good'. They also had to answer 'how hard are the problems', with a similar slider, ranging from 'very easy' to 'very hard'

1.7.1 Cards task

This task was designed to assess participants' tendency to estimate either positive or negative outcomes in the future. In brief, participants had to choose between two cards and were instructed to maximise their points. However, unbeknownst to them, whichever card they chose resulted in a predetermined win or loss. Every ten trials, participants were told how many 'blocks' of ten trials were remaining, and were asked to estimate how many points they would have at the end of the task, and how confident they were in this prediction. We also asked them to estimate their current points, to control for any differences in keeping track of their total score (Supplementary Figure 1A).

We included a block in which participants lost on every trial, allowing us to examine the effect of a run of negative outcomes on future predictions.

1.7.1.1 Exploratory Cards Task Confidence Analyses

We performed two exploratory analyses to examine whether participants' self-rated confidence in the Cards task related to Catastrophizing. Within the Cards task, participants were asked how many points they thought they had won in the previous block, and how many points they would have at the end of the task. Analyses including these variables are presented in the main text. After each of these questions, notably, they were asked how confident they were about their predictions, and we analysed the relationship between each participants' mean confidence rating and their Catastrophizing Questionnaire score using Pearson's correlation.

1.7.2 Mathematics task

This task aimed to measure participants' estimation of their own abilities, in a general context unrelated to a specific stressor or worry, and in such a way that we could control for their actual abilities. We asked participants how good they were at solving mathematical problems, and then asked them to solve such problems, whilst periodically asking them to assess their performance and the difficulty of the problems (Supplementary Figure 1B).

1.8 Deviations From Pre-Registration

In the interest of transparency, we will briefly highlight here the deviations we made from our preregistration (visible at https://doi.org/10.17605/OSF.IO/XRJC5 and in the preregistration folder in https://doi.org/10.17605/OSF.IO/Z2RGK). The most notable deviation is that we state that for certain variables we will use a Spearman's correlation, rather than a Pearson's one, in cases where we expected that data and residuals would be non-normal. Subsequently, we decided to visually inspect residuals for normality, and, if necessary, attempt to transform the data, before resorting to a Spearman's correlation. This deviation was designed to improve our power to detect true effects (reducing our false negative rate).

We also changed how we analysed the effect of 'early burst' balloons in the modified Balloon Analogue Risk Task. We initially planned to calculate the difference in number of pumps before and after the early burst balloons, and relate this to Catastrophizing Questionnaire scores. However, instead we used a mixed effects model, including Catastrophizing Question score and whether the previous trial was an 'early burst' trial as covariates, with a random effect of participant.

We also collected data on the effect of the global COVID-19 pandemic on participants' lives and their levels of worry, in order to attempt to understand the effect that this pandemic had on our data. This data is analysed below.

Due to a technical error, not all participants in the main study were able to complete the high cost block of the modified BART task when it was presented to them as part of the experimental procedure online. These participants were subsequently given the opportunity to complete this block on a later day, but not all chose to do so, resulting in a lower number of participants for this block (242). This also meant that we could not perform the planned exploratory analyses on the effects of block order on risk-taking.

Finally, we performed structural equation modelling rather than multiple regression to test the specificity of our findings – this allows for latent variables to exist that covary with each other, and also allows measurement error to be explicitly accounted for.

2 SUPPLEMENTARY RESULTS

Supplementary Table 2: Demographic information on participants included in both studies

	**Pilot study (n=69)**	**Main study (n=263)**
**Number female (% of sample)**	33 (47.8%)	102 (38.8%)
**Mean age (age range)**	27.5 (range 20-41)	25.5 (range 18-40)
**Number of students (% of sample)**	35 (50.7%)	155 (58.9%)
**Mean prolific score (sd)**	99.2 (1.70)	99.2 (2.24)
**Number with self-reported history of mental illness (% of sample)**	21 (30.4%)	50 (19.0%)

2.1 PILOT STUDY

2.1.1 Cards task

In the Cards task, participants were asked to predict their score at the end of the task. There was no correlation between predicted scores and Catastrophizing scores ($r(67)=-.18$, p =.135; Supplementary Figure 2A). To ensure this estimate was not confounded by individual's memory or their ability to add up points we performed a multiple linear regression analysis where the dependent variable was the participants' estimated final points, and predictors were individual block estimation error (the difference between estimation of points and actual points in each block) and Catastrophizing scores. Neither of these variables significantly predicted participants' predictions of their final points (blockwise estimation error: $\beta=1.00$, $p=.300$; Catastrophizing scores: $\beta=-1.06$, $p=.218$).

Participants were asked how confident they were in both their estimation of the points they won in that block, and their estimation of the total points they would have by the end of the task. In exploratory analyses, we found a significant negative correlation between both of these variables and Catastrophizing scores: (confidence in estimation of points won in current block: $r(67)=-0.29$, p =.017, Supplementary Figure 2b; confidence in estimation of total points at end of task: $r(67)=-0.41$, $p=0.0004$, Supplementary Figure 2c). Participants with higher Catastrophizing scores reported being less confident in their estimations of points.

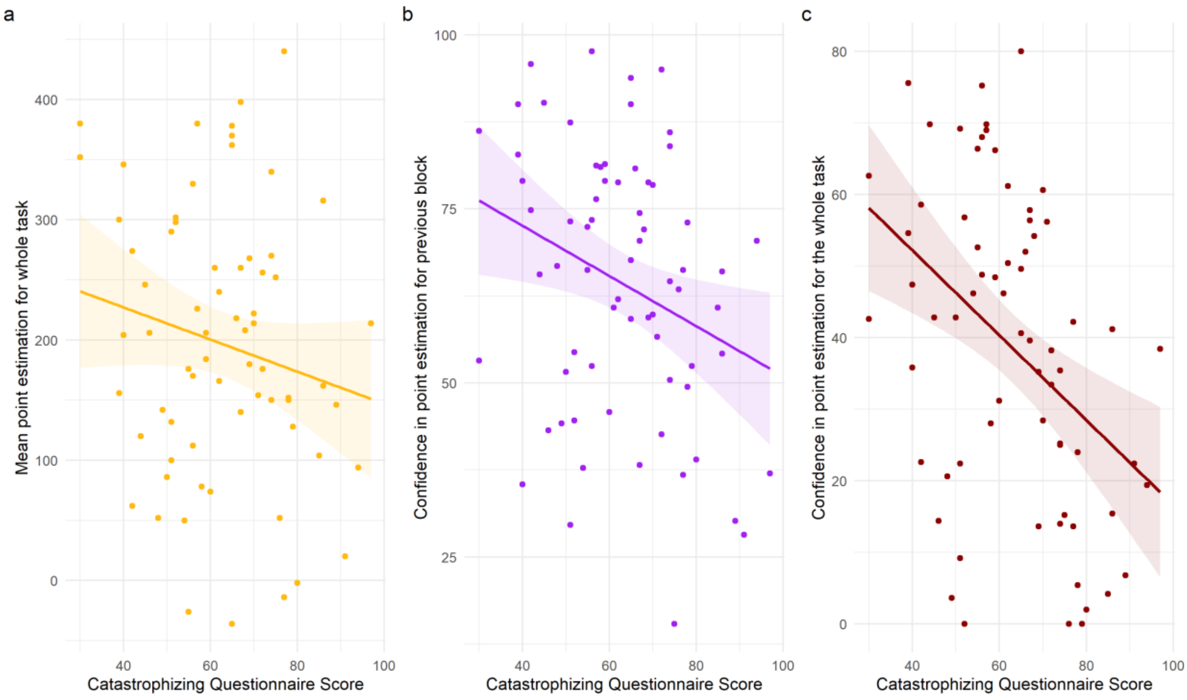

Supplementary Figure 2: Results of analyses of Cards task. Scatter plot and regression line for relationship between Catastrophizing Questionnaire score and a) the mean of participant's estimation of the total points they would have by the end of the task, b) participants' self-rated confidence in their estimation of the points they won in the previous block, and c) their confidence in their estimation of how many points they would have at the end of the task.

2.1.1.1 Discussion

Overall, we found no evidence that catastrophizing is related to the prediction of negative outcomes in a task in which participants selected cards, and then received or lost points. This may indicate that the prediction of negative outcomes only occurs in emotionally charged or stressful situations, rather than being a more general process that occurs in response to uncertainty in general. However, in an exploratory analysis we did find that there might be a relationship between catastrophizing and confidence, which requires further replication, perhaps using existing metacognition paradigms.

2.1.2 Mathematics task

In the mathematics task, we examined whether there was a relationship between participants' pre-task subjective estimates of their ability to solve maths problems and their Catastrophizing scores. Participants with higher catastrophizing scores reported that their ability to solve maths problems was lower than those with low catastrophizing scores (Spearman's rank test $r_s(67)=-0.37$, $p=.001$; Supplementary Figure 3A).

The effect of Catastrophizing scores on pre-task subjective estimated ability was no longer significant in a multiple linear regression analysis, in which Catastrophizing scores, ongoing subjective estimation of performance, ongoing estimation of problem difficulty, and actual (objective) performance were allowed to predict the dependent variable, pre-task subjective estimated ability (Supplementary Figure 3B). The only significant predictor in this model was subjective performance ($\beta=0.69$, $p=.0007$), and Catastrophizing score was no longer a significant predictor ($\beta=-0.35$, $p=.071$). This model fit the data well ($F_{4,64}=10.25$, $p<.0001$, $R^2=0.391$).

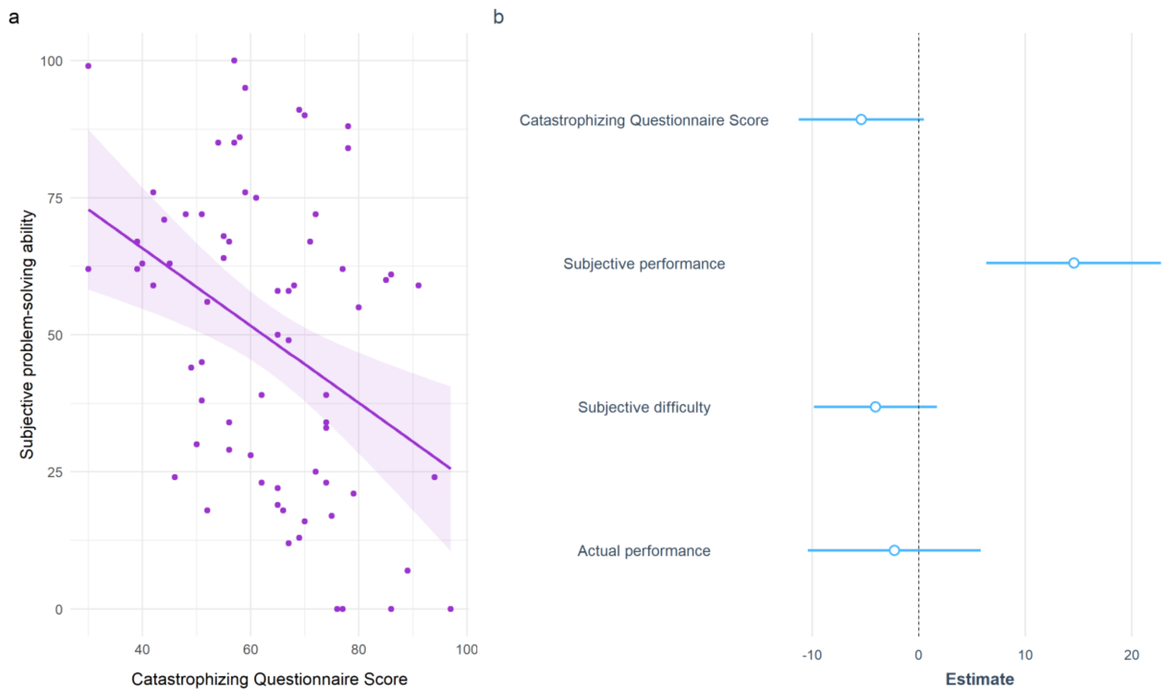

Supplementary Figure 3: Results of analyses of Mathematics task. A) Scatter plot and regression line for relationship between Catastrophizing Questionnaire score and participants' self-rated subjective ability to problem-solve. B) However, when entered into a multiple regression including subjective performance, subjective difficulty of problems and actual performance, there was no longer a significant effect of catastrophizing.

2.1.2.1 Discussion

In summary, we found evidence that catastrophizing might be related to subjective self-reported ability in the Mathematics task, although this evidence was attenuated when we controlled for ongoing estimation of performance. However, this attenuation may be because ongoing estimation of performance and subjective ability are measuring the same latent variable (notably, there is a significant correlation between these two variables in the multiple regression).

Further work could investigate how this relates to our exploratory finding from the Cards task that self-reported confidence in judgements is related to catastrophizing – both of these measures, whilst obtained for separate tasks, probe an individual's (mathematics-related) self-efficacy and metacognition.

2.1.3 BART task

2.1.3.1 Computational analysis

The best-fitting model for the pilot dataset was the classic four-parameter model, with a prior belief about how many times a balloon could be pumped up before bursting, a learning rate, an inverse temperature parameter, and a risk-taking parameter. The total integrated BIC scores are shown in Supplementary Figure 4.

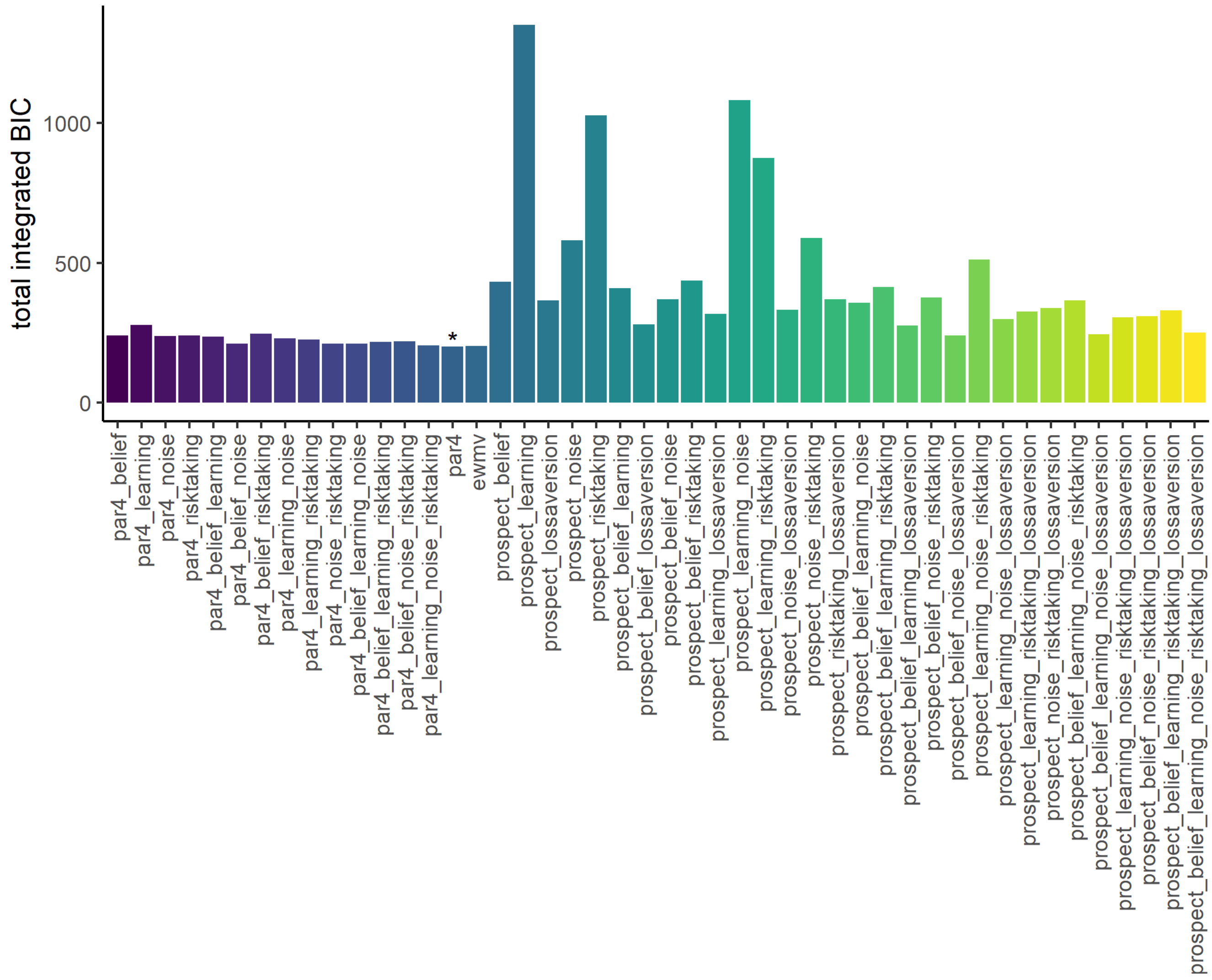

Supplementary Figure 4: Integrated BIC scores for BART models fit to the pilot dataset. Note that lower total integrated BIC indicates a better fit. Here, 'par4' refers to hBayesDM's 4 parameter model, 'ewmv' refers to the exponential-weighted mean-variance model, 'prospect' refers to our novel prospect-theory based models, and all other models are variations of the 'par4' model, including subsets of the parameters in this full model. Here, 'belief' refers to the prior belief of how many pumps it will take before a balloon bursts, 'learning' refers to a learning rate, 'noise' to an inverse temperature term, 'risktaking' to the risk taking parameter, and 'lossaversion' to the loss aversion parameter. The asterisk denotes the best-fitting model.

To test parameter recovery, we used fitted parameters from 100 of the participants completing the 'low cost' block of the task.

We fit choices generated using these parameters using RStan and hBayesDM, with 2000 iterations and a single chain. We display how veridically the model can recover the synthetic parameters (diagonal: high numbers indicate high correlation between synthetic and recovered parameters), and how correlated the recovered parameters are with each other (off-diagonal: low numbers indicate tradeoff).

The recovery of each parameter was good, with minimal tradeoff between parameters (Supplementary Figure 5).

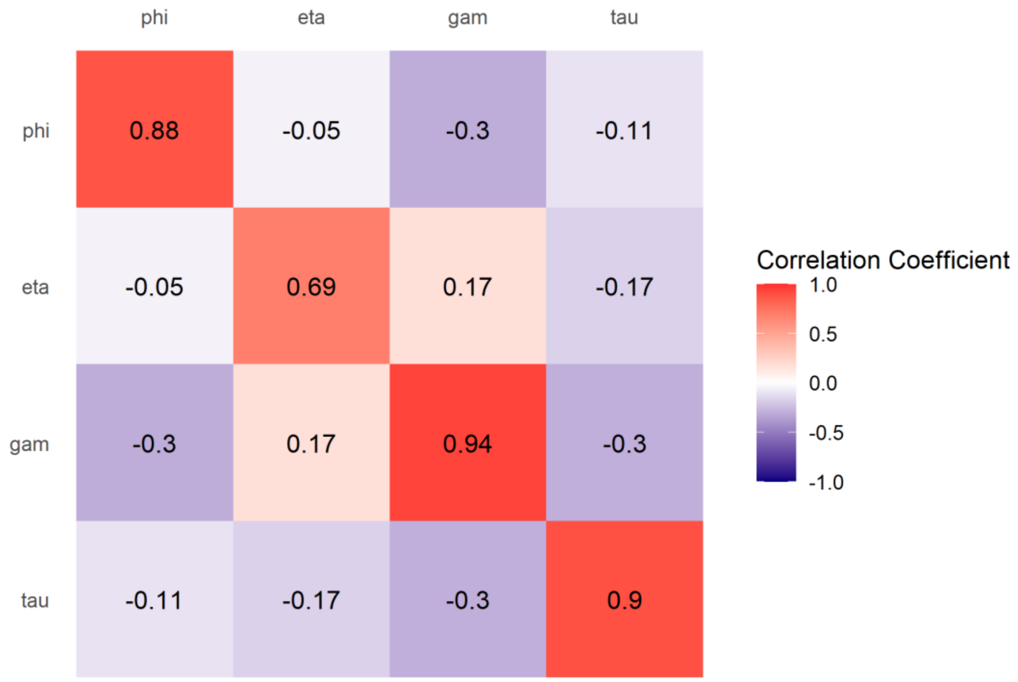

Supplementary Figure 5: A confusion matrix showing that the parameters from the best-fitting model can be recovered. The diagonal represents correlation coefficients between synthetic parameters and recovered parameters (after fitting using hBayesDM), and the off-diagonal represents the correlation between these fitted parameters.

To show convergence, we also inspected the traceplots of the log posterior (Supplementary Figure 6). We also examined the R-hat values of the best fitting models (Supplementary Figure 7), a measure of convergence, where Rhat<1.1 indicates that the chains have probably converged, and <1.01 indicates that they almost certainly have (Gelman & Rubin, 1992; Vehtari, Gelman, Simpson, Carpenter, & Bürkner, 2021).

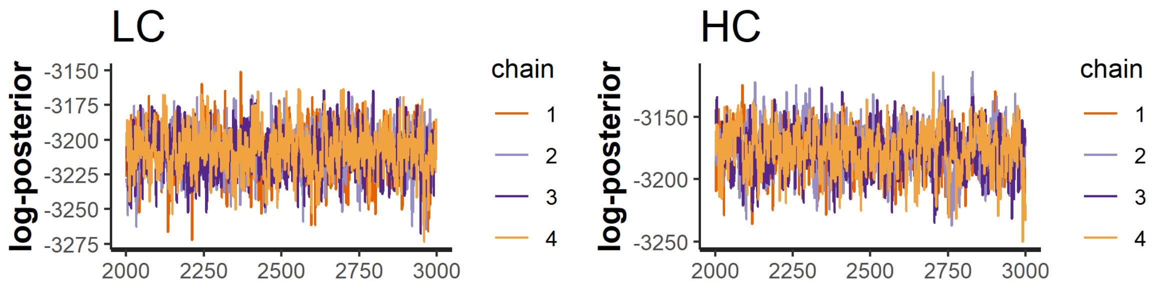

Supplementary Figure 6: Traceplots of the best-fitting models, showing convergence of the multiple chains.

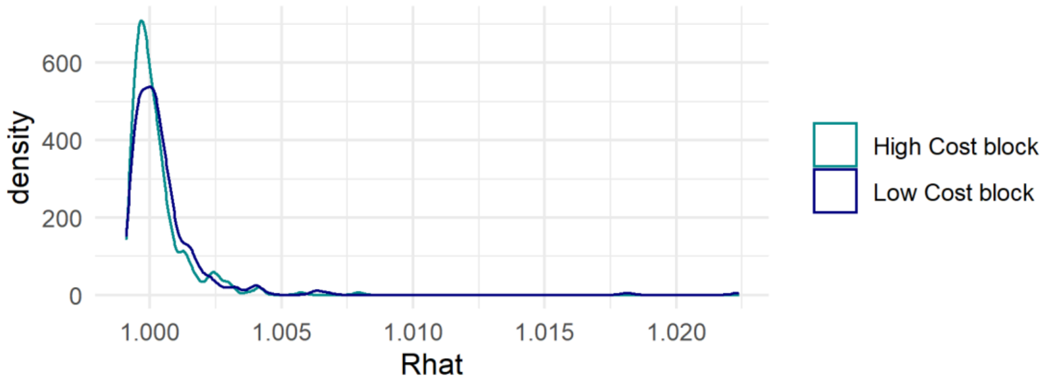

Supplementary Figure 7: Histogram of R-hat values for the best-fitting model in both the high and low cost blocks.

The results of correlation analyses between each of these variables and Catastrophizing Questionnaire scores are shown in Supplementary Table 3.

**Variable**	**Block**	***r***	***p***
Prior belief	Low Cost	0.227	0.061
	High Cost	0.121	0.321
Learning rate	Low Cost	-0.087	0.476
	High Cost	0.246	0.042
Inverse temperature	Low Cost	-0.138	0.258
	High Cost	-0.069	0.572
Risk-taking	Low Cost	-0.211	0.082
	High Cost	-0.147	0.227

Supplementary Table 3: Correlations between Catastrophizing Questionnaire scores and variables from the best-fitting model, per block. The correlation coefficient and p values are shown. For all the above correlations, there are 67 degrees of freedom.

2.1.3.2 Early burst balloons

In the HC block, for some balloons, known as “early burst balloons”, the number of pumps needed to burst the balloon was deliberately low (between 1 and 3 pumps; the fact that there were “early burst balloons” was unknown to the participants) so we could assess how this inevitable outcome would affect participants’ willingness to pump the balloon up on subsequent trials. We examined the effect of ‘early burst’ balloons by performing a mixed regression analysis, including Catastrophizing score and whether the previous trial was an ‘early burst’ trial as covariates, with a random effect of participant. In this model there was no significant effect of the presence of an ‘early burst’ balloon on the previous trial (β=0.286, p=.083), nor was there a significant effect of Catastrophizing scores (β=-0.0009, p=.817), nor any interaction between the two (β=-0.004, p=.167).

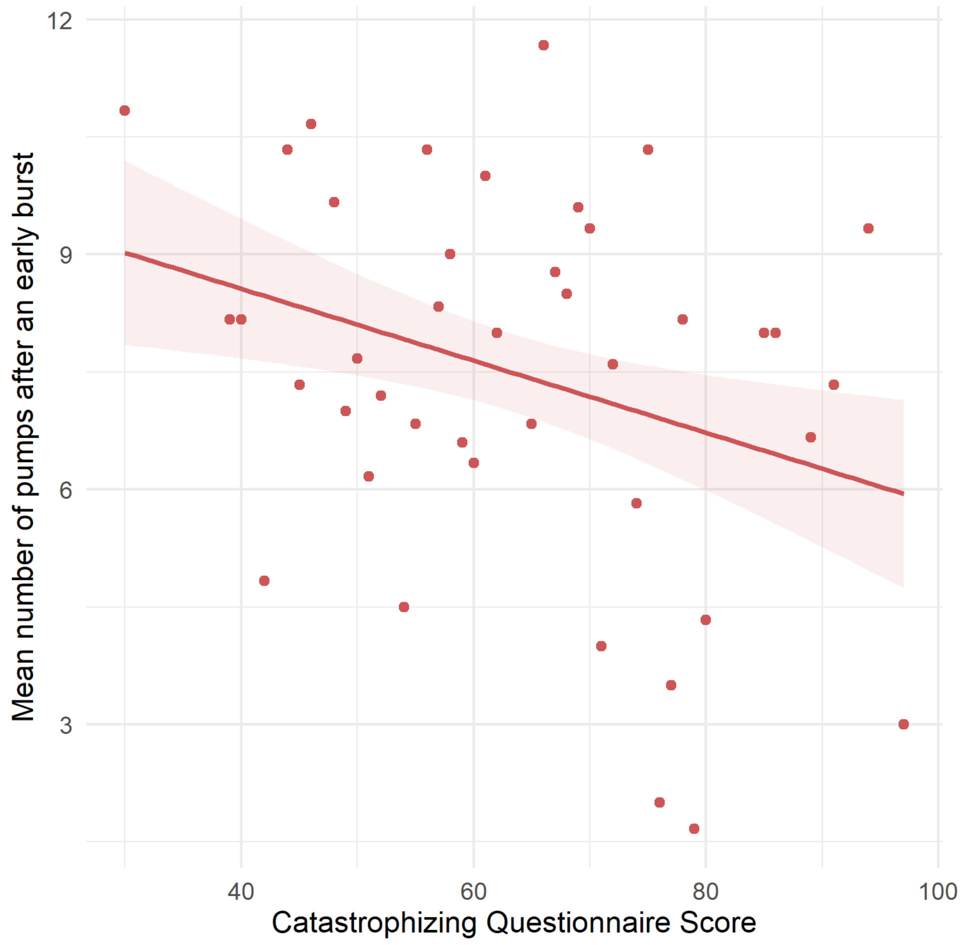

Supplementary Figure 8: There was no significant relationship between the number of times each participant pumped the balloon up on the trial after an 'early burst' balloon in the BART task and their Catastrophizing scores.

2.2 Main Study

2.2.1 Data inspection

There was no floor effect in either block. One-sided t-tests indicated that the mean number of pumps in each block (before any participants were removed) was significantly different from 0 (LC: M=8.366, SD=2.36, $t(264)$=57.756, p<0.001; HC: M=7.09, SD=2.64, $t(248)$=42.322, p<0.001; Supplementary Figure 9).

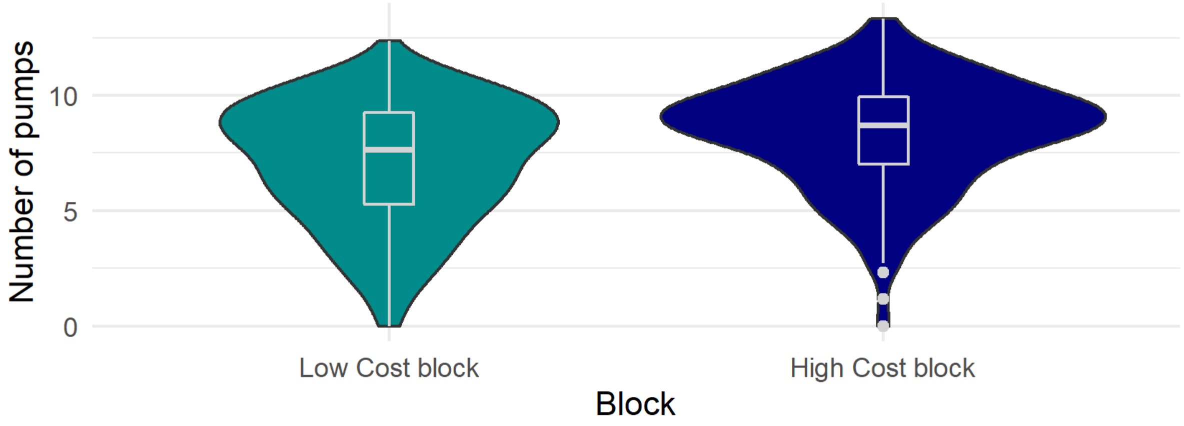

Supplementary Figure 9: Distribution of number of pumps in the Low Cost and High Cost blocks.

2.2.2 Structural equation modelling

2.2.2.1 Mediation analysis

To test the hypothesis that catastrophizing mediates a relationship between anxiety and risk-aversion, we performed an exploratory mediation analysis. We used anxiety as the independent variable (a latent variable, comprised of participants' scores on the three anxiety-relevant questionnaires: the PSWQ, STAI-T, and GAD-7), Catastrophizing scores as the mediator, and transformed number of pumps in the LC block as the outcome variable (representing risk-aversion here).

The mediation analysis was inconclusive, probably as there was no relationship between anxiety and risk-taking (operationalised as transformed number of pumps in the LC block: β=-0.001, *SE*=0.007, *p*=0.924). There was a significant relationship between anxiety and catastrophizing (β=1.314, *SE*=0.061, *p*<0.001), but no significant relationship between catastrophizing and the number of pumps, when anxiety was controlled for (β=0.005, *SE*=0.004, *p*=0.248). The indirect relationship was not significant (β=0.006, *SE*=0.006, *p*=0.247): the bias-corrected bootstrapped confidence interval with 10,000 samples crossed 0: 95% CI [-0.005, 0.017]. However, the overall total effect was significant (β=0.006, *SE*=0.003, *p*=0.041).

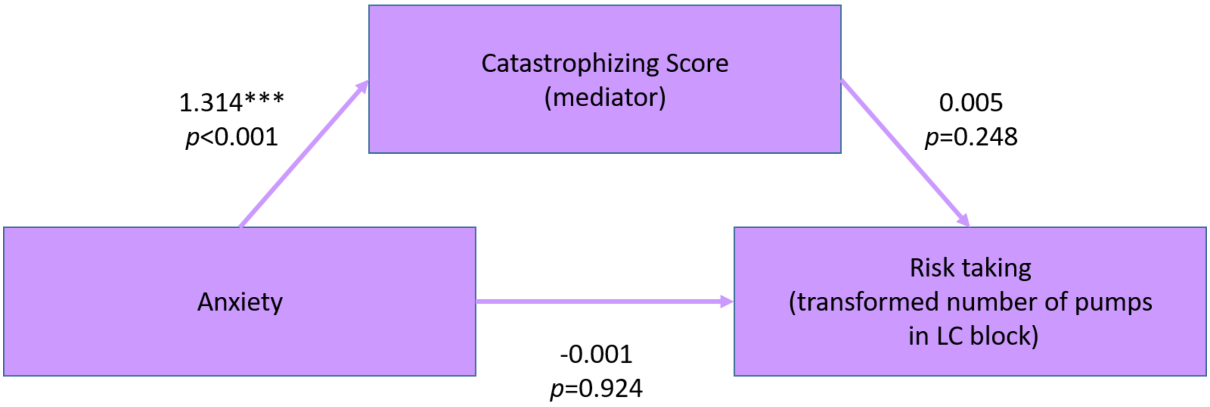

Supplementary Figure 10: Diagram of mediation analysis. Here, anxiety is the independent variable, made from summing scores of three different anxiety questionnaires, catastrophizing score is the mediator, and risk taking is the outcome variable. The direct relationship is not significant, although both of the components of the indirect pathway are (represented with asterisks).

2.2.3 Structural equation modelling analysis

To examine the specificity of the relationship between catastrophizing and number of pumps in the LC block, we created an exploratory structural equation model with several latent factors: catastrophizing (comprised of Catastrophizing questionnaire scores), anxiety (comprised of PSWQ, GAD-7 and STAI scores), depression (comprised of PHQ-8 scores) and risk-taking (comprised of the transformed mean number of pumps in the LC block). We allowed catastrophizing, anxiety and depression to covary, and specified a regression of catastrophizing onto risk-taking (see Supplementary Figure 11 for the resulting path diagram).

This model, in which catastrophizing alone was regressed against risk-taking, had generally adequate fit statistics ($T(8)=47.18$, $p<0.001$; CFI=0.962, TLI=0.930, RMSEA=0.136[0.100-0.175], SRMR=0.029, BIC= 8933). The regression between catastrophizing and risk-taking in this model was significant (standardized $\beta=-0.125$, $p=.042$). A nested model with regressions between anxiety and risk-taking and depression and risk-taking (in addition to the regression between catastrophizing and risk-taking, see red arrows on Supplementary Figure 11) did not provide a better fit to the data, providing some evidence for specificity of the effect to catastrophizing ($\chi^2=0.366$, $p=.833$, BIC=8943).

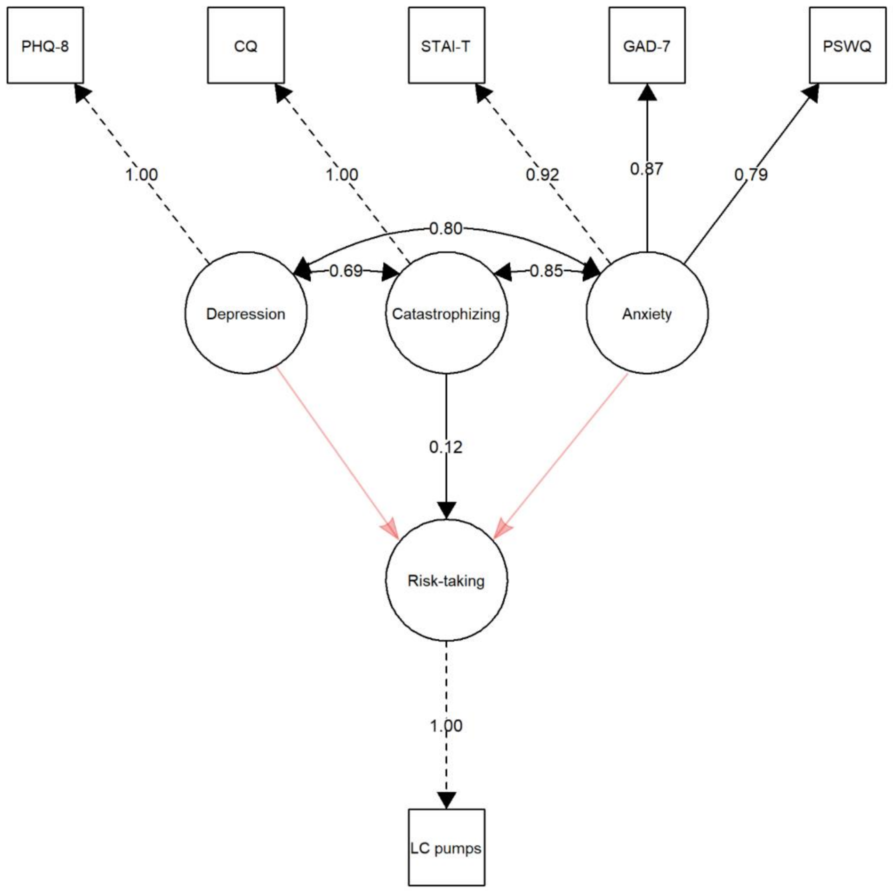

Supplementary Figure 11: Path diagram of the structural equation model fit to examine the specificity of the relationship between catastrophizing and risk-taking. Ellipses represent latent variables, and rectangles represent indicator (or measured) variables. Single-headed arrows represent regressions, and double-headed arrows represent covariances. For simplicity, we do not display the residual variances here. Fixed parameters are represented with dashed lines. The structural equation model includes covariance between depression, catastrophizing and anxiety, and a regression of risk-taking on catastrophizing. Standardized parameter estimates are displayed on the edges. Note that the regression between risk-taking and catastrophizing is statistically significant. A second model, with additional regressions between depression and risk-taking and anxiety and risk-taking (displayed in red, or with pale dotted lines in print version, and not included in the main model), was not a significant improvement over the main model.

2.2.4 Computational analyses

The best-fitting model for the main dataset was the classic four-parameter model, which had a prior belief about how many times a balloon could be pumped up before bursting, and a risk-taking parameter. The total integrated BIC scores are shown in Supplementary Figure .

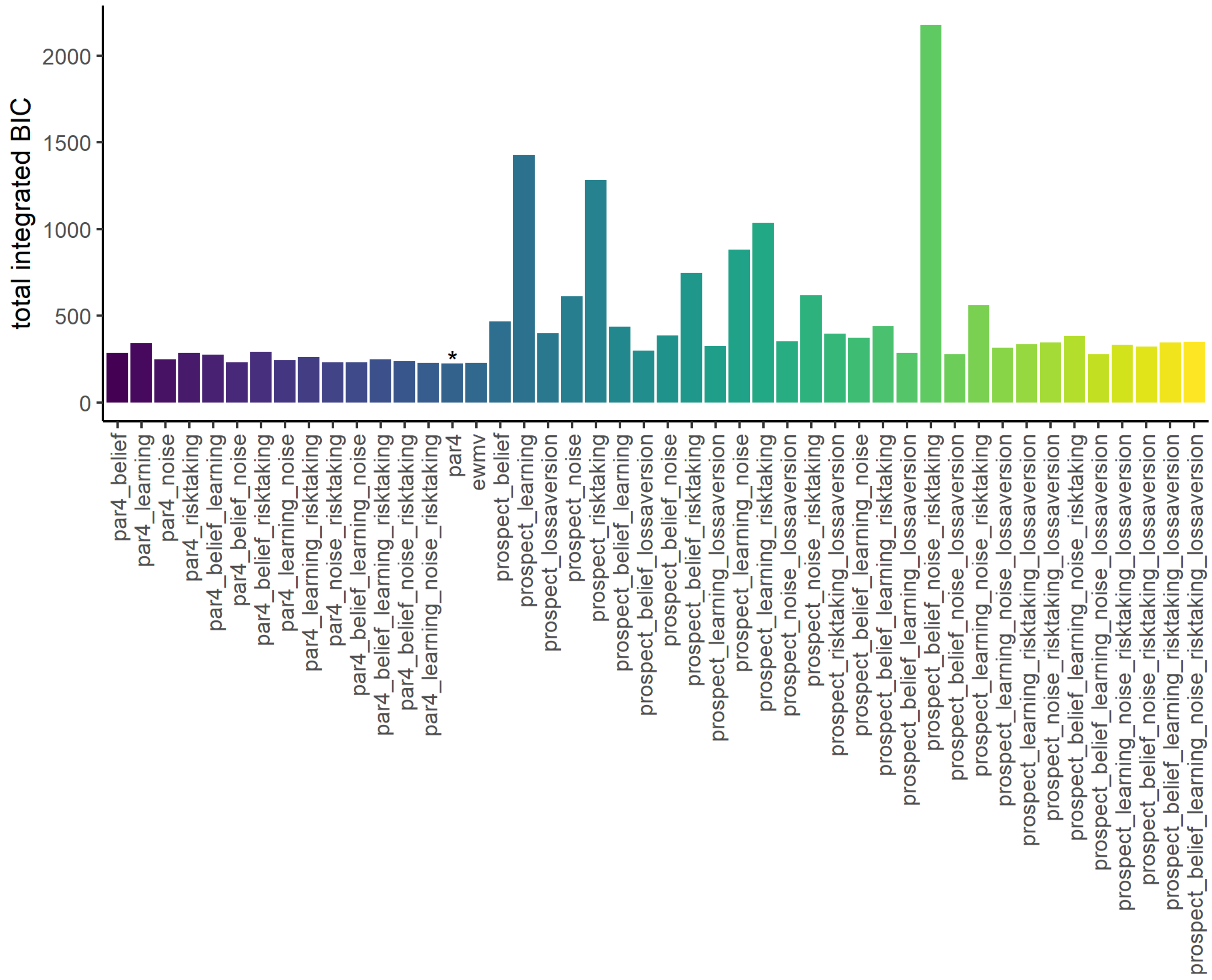

Supplementary Figure 12: Integrated BIC scores for BART models fit to the main dataset. Note that lower total integrated BIC indicates a better fit. Here, 'par4' refers to hBayesDM's 4 parameter model, 'ewmv' refers to the exponential-weighted mean-variance model, 'prospect' refers to our novel prospect-theroy based models, and all other models are variations of the 'par4' model, including subsets of the parameters in this full model. Here, 'belief' refers to the prior belief of how many pumps it will take before a balloon bursts, 'learning' refers to a learning rate, 'noise' to an inverse temperature term, 'risktaking' to the risk taking parameter, and 'lossaversion' to the loss aversion parameter. The asterisk denotes the best-fitting model.

Parameter recovery for this model can be seen above, in the results for the computational analysis of the pilot data, as the same model fit the data best in each case (Supplementary Figure 5).

To show convergence, we also present the traceplots of the log posterior (Supplementary Figure) and a histogram of R-hat values (Supplementary Figure).

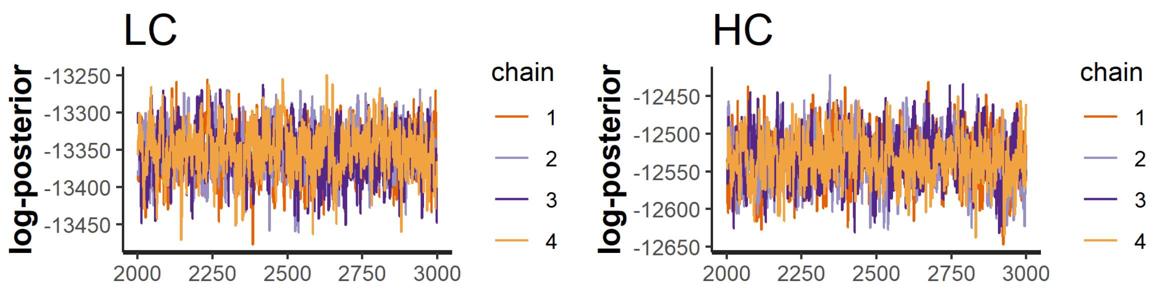

Supplementary Figure 9: Traceplots of the best-fitting model for both task versions, showing convergence of the multiple chains.

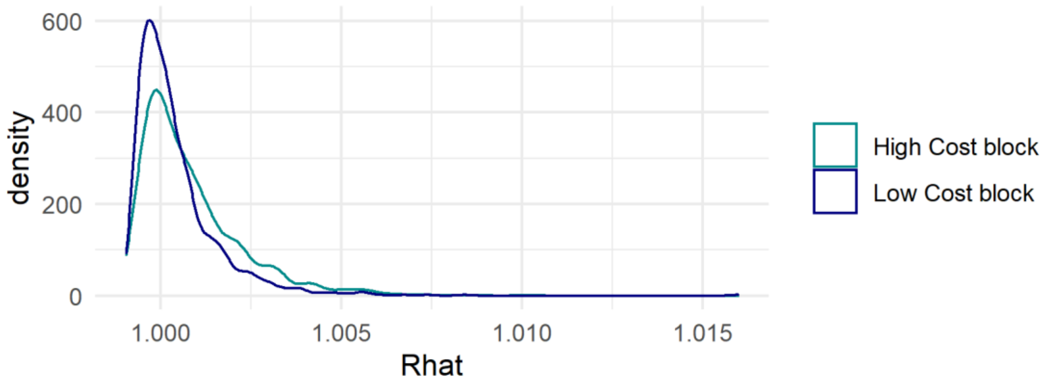

Supplementary Figure 10: Histogram of R-hat values for the best-fitting model in both the high and low cost blocks.

The results of correlation analyses between each of these variables and Catastrophizing Questionnaire scores are shown in Supplementary Table 4.

**Variable**	**Block**	***r***	***p***
Prior belief	Low Cost	0.054	0.405
	High Cost	-0.111	0.088
Learning rate	Low Cost	-0.035	0.590
	High Cost	0.006	0.932
Inverse temperature	Low Cost	-0.044	0.496
	High Cost	0.001	0.988
Risk-taking	Low Cost	-0.016	0.809
	High Cost	0.037	0.575

Supplementary Table 4: Correlations between Catastrophizing Questionnaire scores and variables from the best-fitting model, per block. The correlation coefficient and p values are shown. For all the above correlations, there are 237 degrees of freedom.

2.2.5 Early burst balloons BART task

In the version of the task we used in the main study, we included early burst balloons in both blocks, rather than just in the HC block. In a mixed model, with Catastrophizing scores and the occurrence of an 'early burst' balloon (both blocks) on a previous trial as predictors of per-trial balloon pumps, there were no statistically significant main effects or interactions, in either block.

2.3 COVID-19 Pandemic

Our results indicate that the participants with higher catastrophizing scores tend to also score higher in the COVID-19 impact questionnaire items that asked about worry (pilot study: $r(67)=0.27$, $p=0.025$; main study: $r(261) = 0.17$, $p=.007$). However, we recruited participants for our pilot study for whom we had baseline catastrophizing data (Pike et al., 2021), and found, using a paired samples t-test, that Catastrophizing scores did not change significantly between July 2019 and April 2020 ($t(68)=-0.88$, $p=0.380$). The significant positive correlation between the catastrophizing scores and the worry about the pandemic, and lack of difference between the catastrophizing scores before and during the pandemic, indicates that the results gathered in the present study were not directly influenced by the pandemic and therefore are reliable and reproducible.

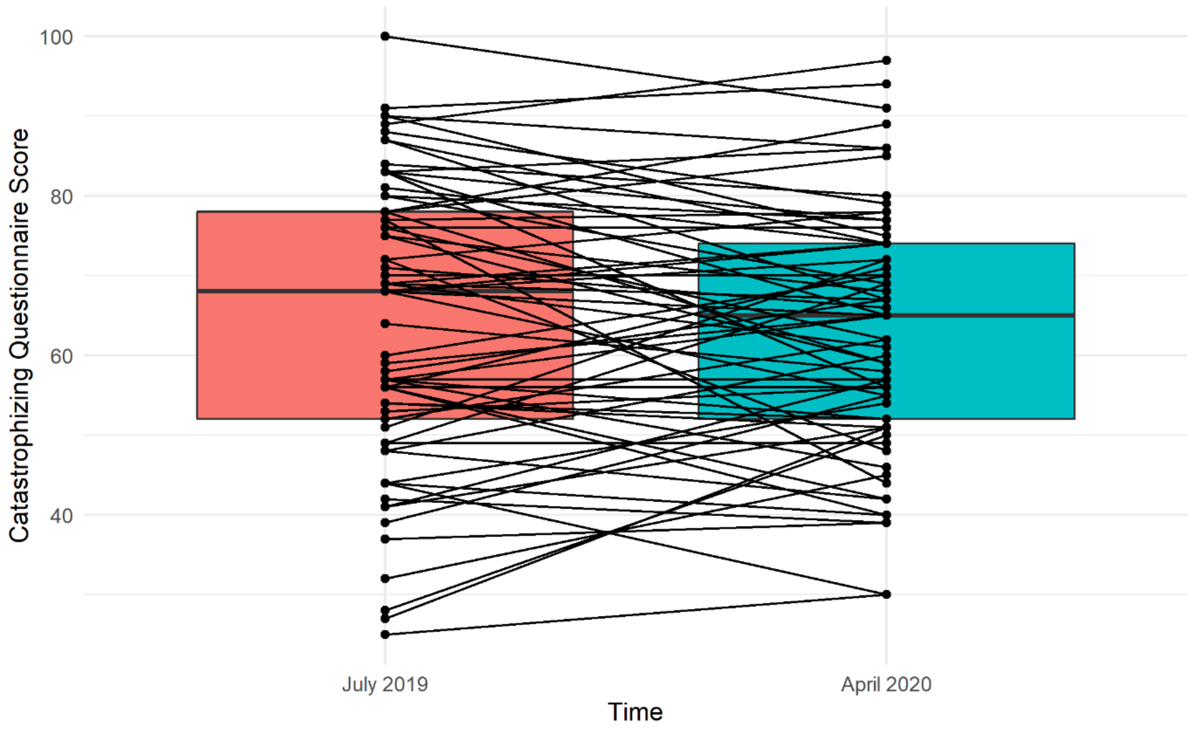

Supplementary Figure 11: Boxplots with overlaid points and within-participant lines joining them for Catastrophizing Questionnaire scores obtained from the same participants in July 2019 (pre COVID-19 pandemic) and April 2020 (after the pandemic had started).

3 Additional References

Gelman, A., & Rubin, D. B. (1992). Inference from iterative simulation using multiple sequences (with discussion). *Statistical Science*, *7*(4), 457–511.

Pike, A. C., Serfaty, J. R., & Robinson, O. J. (2021). The development and psychometric properties of a self-report Catastrophizing Questionnaire. *Royal Society Open Science*, *8*(1), 201362. doi:10.1098/rsos.201362

Vehtari, A., Gelman, A., & Gabry, J. (2017). Practical Bayesian model evaluation using leave-one-out cross-validation and WAIC. *Statistics and Computing*, *27*(5), 1413–1432. doi:10.1007/s11222-016-9696-4

Vehtari, A., Gelman, A., Simpson, D., Carpenter, B., & Bürkner, P.-C. (2021). Rank-Normalization, Folding, and Localization: An Improved $\hat{R}$ for Assessing Convergence of MCMC (with Discussion). *Bayesian Analysis*, *16*(2). doi:10.1214/20-BA1221
